# Navigating Therapeutic Challenges of Pyoderma Gangrenosum in Felty's Syndrome: A Case Report and Literature Review

**DOI:** 10.7759/cureus.71428

**Published:** 2024-10-14

**Authors:** Victor D Acuña-Rocha, Gerardo Sanchez Solís, Jose A Ramírez-Vázquez, Anette Fischer Rouyer, Iván de Jesús Hernández Galarza

**Affiliations:** 1 Internal Medicine, Hospital Universitario Dr. José Eleuterio González, Monterrey, MEX; 2 Rheumatology, Hospital Universitario Dr. José Eleuterio González, Monterrey, MEX

**Keywords:** dapsone, dapsone treatment, felty's syndrome, pyoderma gangrenosum, rheumatoid arthritis

## Abstract

Pyoderma gangrenosum (PG) is a rare neutrophilic dermatosis characterized by painful skin ulcers. Treatment typically involves systemic corticosteroids, calcineurin inhibitors, or tumor necrosis factor-alpha inhibitors. Currently, treatment guidelines are not well established, making it important to consider alternative options in complicated cases. We report the case of a 52-year-old female with rheumatoid arthritis (RA) and a history of Felty's syndrome (FS) who developed PG and subsequently developed neutropenia due to azathioprine. The patient achieved remission with the use of dapsone and filgrastim. This treatment may be effective as an alternative for patients with RA and FS.

## Introduction

Pyoderma gangrenosum (PG) is a rare neutrophilic dermatosis (ND) characterized by painful skin ulcers with rapid evolution, undermined edges, and peripheral erythema. It represents a diagnostic challenge that demands a classical clinical presentation and biopsies to rule out other significant pathologies. Delayed or incorrect diagnosis may result in considerable morbidity [1,2]. Between 50% and 70% of cases are associated with some systemic disease such as inflammatory bowel disease (20.2%), rheumatoid arthritis (RA) (11.8%), hematologic malignancies (3.9%), and solid tumors (7.4%) [2]. Treatment generally involves systemic corticosteroids, calcineurin inhibitors, or tumor necrosis factor-alpha inhibitors, as these drugs have shown the most efficacy [3]. However, due to the lack of established clinical guidelines, it is crucial to consider alternative treatments in complex cases.

In this report, we present the case of a 52-year-old female with rheumatoid arthritis (RA) and a history of Felty's syndrome (FS) who developed PG. Her condition was complicated by neutropenia caused by azathioprine. She achieved remission with the administration of dapsone and filgrastim, suggesting that this combination may be an effective alternative treatment for patients with RA and FS.

## Case presentation

**Figure 1:**
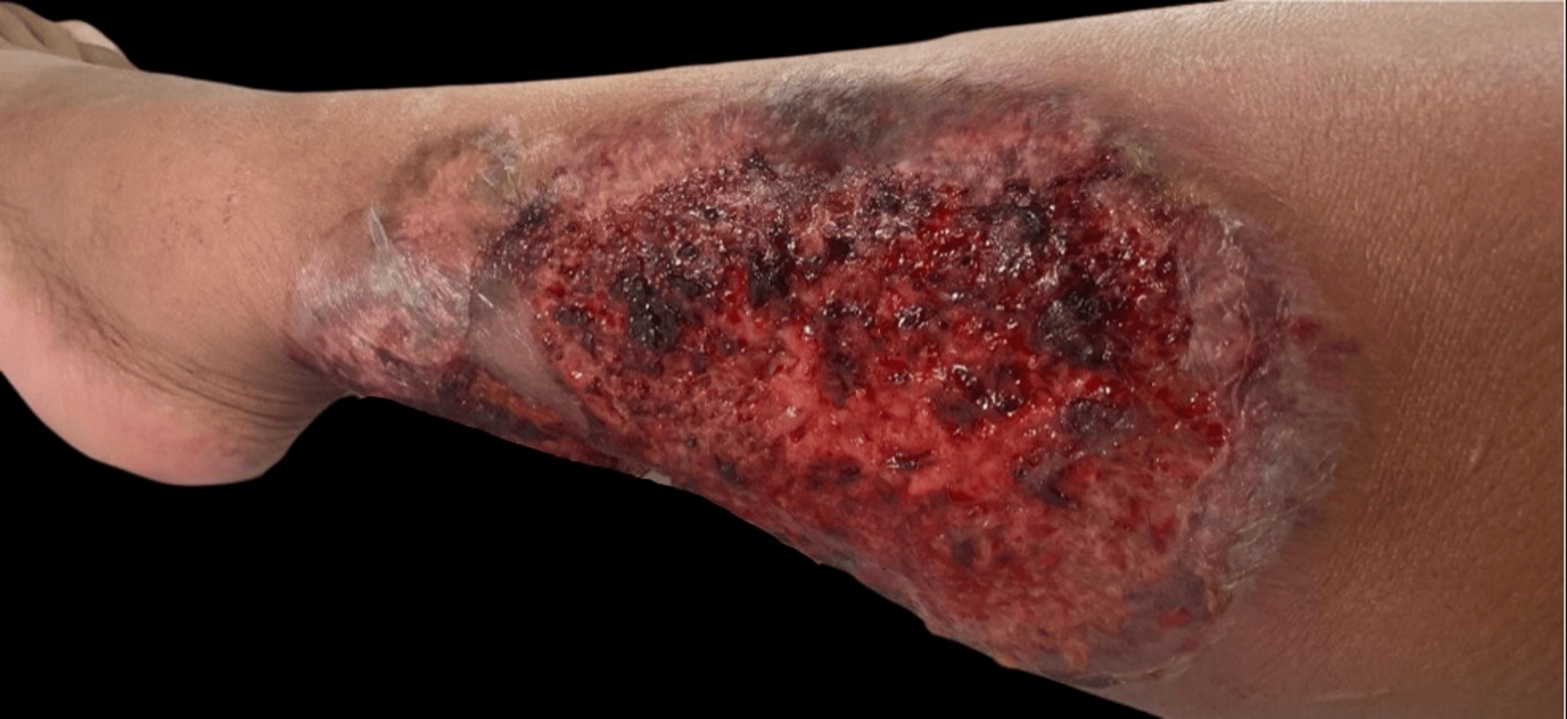
Upon starting dapsone Ulcer on the left anterolateral region measuring 22 cm in diameter, characterized by irregular, non-grouped borders with intensely erythematous peripheral halos and necrotic eschar.

We present the case of a 52-year-old female patient with a 12-year history of rheumatoid arthritis in remission, managed with methotrexate, and Felty's syndrome, currently not under treatment. Her current condition began two months prior to admission, with the appearance of tense, violaceous blisters on the anterior region of both legs, measuring 3 cm in diameter and associated with continuous stabbing pain rated as 3/10 on the visual analog scale (VAS), worsening with palpation. One month before admission, the lesions increased in size and developed erythematous borders, and the pain worsened to 5/10 on VAS. At this point, a biopsy of the lesions was performed, along with potassium hydroxide (KOH) preparation and wound cultures. Eight days before admission, the lesions ulcerated and expanded over the surface of both legs, accompanied by serosanguineous discharge and progressive pain, intensifying to 9/10 on the VAS. Upon arrival at the emergency department, the patient was in good general condition. A skin examination revealed an ulcer on the left anterolateral region, measuring 22 cm in diameter, with irregular, scattered edges, marked erythematous halos, and areas of necrosis (Figure 1).

**Figure 2:**
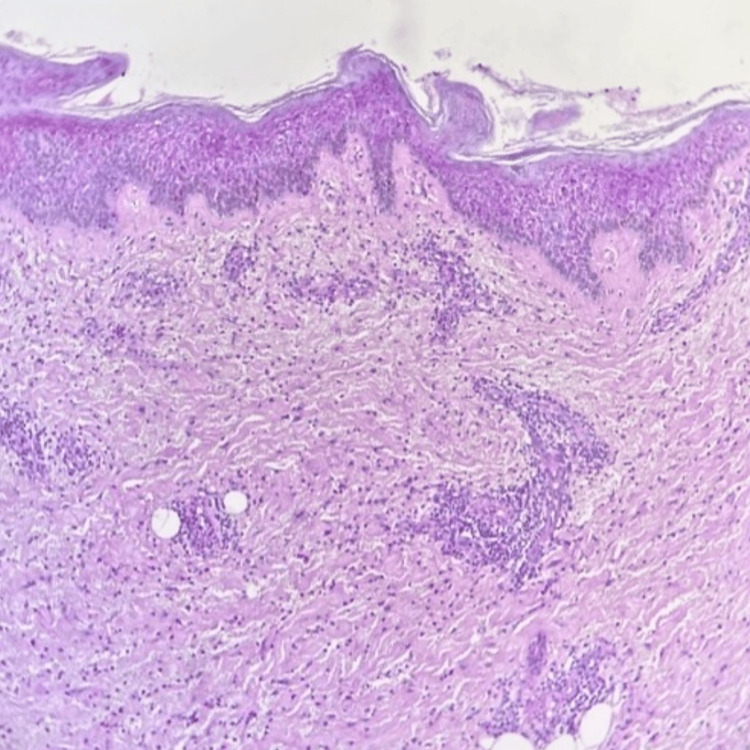
Pathological findings (hematoxylin and eosin staining) The scale bar represents 500 μm. It depicts neutrophilic infiltration indicative of an acute inflammatory process affecting the epidermis and extending into the subcutaneous tissue.

Laboratory tests upon admission showed a normal biochemical profile, normocytic normochromic anemia, leucocyte count of 4.11 K/uL, neutrocyte count of 3.07 K/uL, erythrocyte sedimentation rate (ESR) of 39 mm/hour, and C-reactive protein (CRP) of 13.8 mg/dL. Biopsy revealed a neutrophilic infiltration compatible with an acute inflammatory process involving the epidermis to the subcutaneous tissue. Areas of necrosis and ulceration are evident, along with signs of edema and fibrin deposition consistent with pyoderma gangrenosum (Figure 2). KOH, Ziehl-Neelsen stain, and cultures were negative. Management started with non-steroidal anti-inflammatory drugs (NSAIDs) for analgesia, topical clobetasol solution, topical mupirocin, and oral azathioprine 100 mg/day. Methotrexate was discontinued upon the recommendation of rheumatology.

Five days later, new studies showed CRP of 0.5 mg/dL and ESR of 42 mm/hour, as well as mild neutropenia of 0.99 K/uL, associated with azathioprine, which was discontinued and replaced with oral dapsone 50 mg every 24 hours, with filgrastim added, resulting in neutrophil count elevation to 8.24 K/uL two days later. The patient clinically improved, with pain resolution and no evidence of infection, leading to medical discharge for outpatient management with oral dapsone 50 mg every 24 hours, topical clobetasol 0.05% solution applied every 12 hours, and oral prednisone 60 mg every 24 hours and oral hydroxychloroquine 400 mg every 24 hours at night, along with wound care.

**Figure 3:**
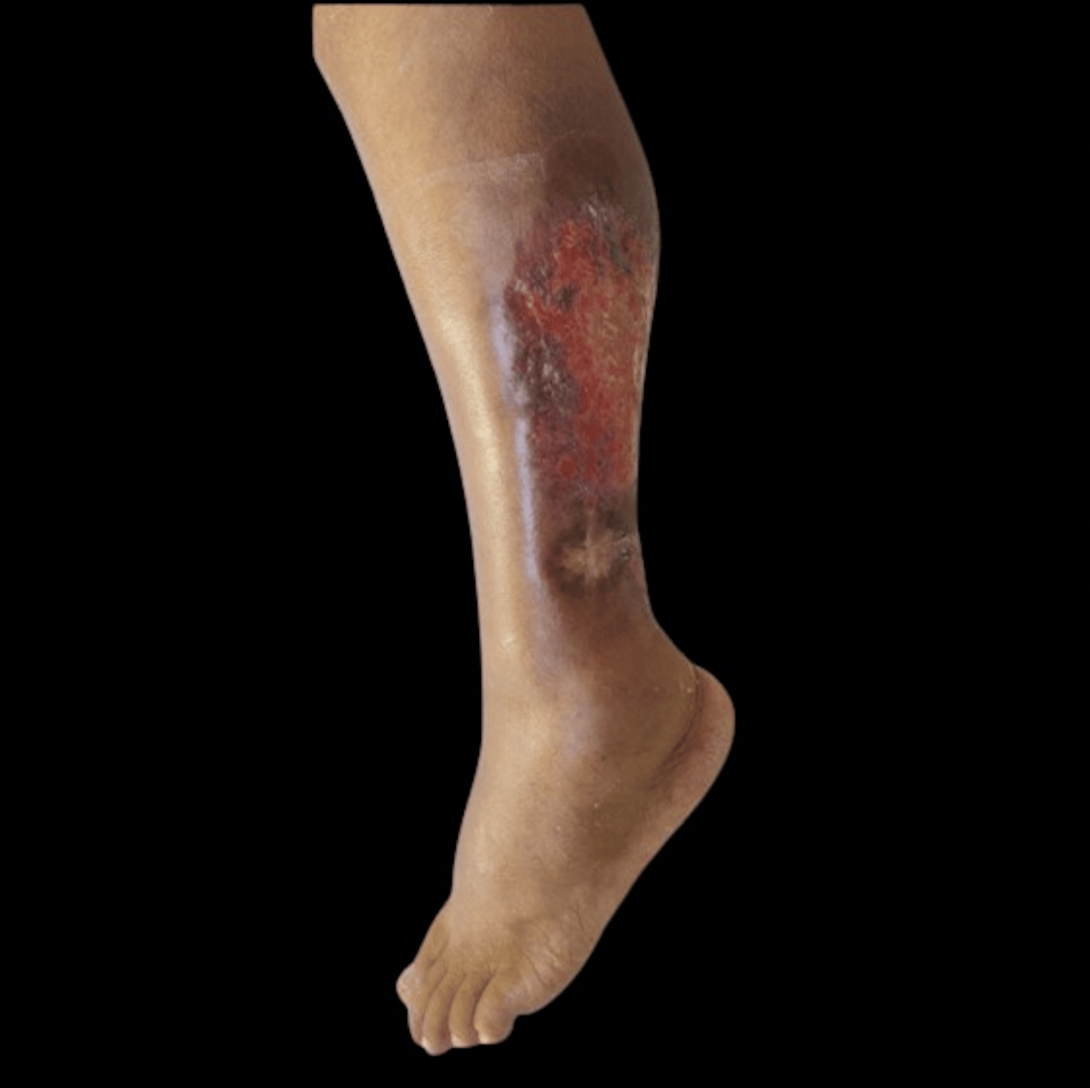
At one-month follow-up after starting dapsone Evolution of the skin lesion over time.

During the one-month follow-up, significant improvement was noted in lesions with decreased ulceration and erythematous appearance (Figure 3).

## Discussion

Pyoderma gangrenosum is a rare cutaneous manifestation, generally associated with an underlying disease, most frequently with inflammatory bowel disease and, secondly, with joint diseases, with rheumatoid arthritis being the most reported, found in 8.7%-11.8% of cases [1,2].

Currently, the treatment for this disease has changed due to the discovery of new targeted immunomodulatory therapies; however, systemic or topical corticosteroids are still the first line of treatment, as well as calcineurin inhibitors, either topical or systemic. The second line of treatment includes tumor necrosis factor-alpha inhibitors, dapsone, mycophenolate mofetil, or interleukin (IL)-1 and IL-17 inhibitors [3]. Although treatment usually begins with the administration of systemic corticosteroids due to their rapid immunosuppressive action [3], the decision to initiate a corticosteroid-sparing treatment in our case, due to the patient's history of previous treatment, led to the use of azathioprine as an immunosuppressive treatment.

One of the most frequently reported side effects of azathioprine use is myelotoxicity, characterized mainly by leukopenia, with an incidence of 5%-25% [4]. Literature reports indicate that genetic variants, primarily in the enzymes thiopurine S-methyltransferase (TPMT) and nudix hydrolase 15 (NUDT15), are associated with an increased risk of developing myelosuppression, conferring up to twice the risk [5].

In our case, the patient developed moderate neutropenia, which was not documented at the time of admission in laboratory tests and therefore was not attributed to the history of Felty's syndrome. Given the patient's underlying diagnosis of Felty's syndrome and the risk of infections associated with myelotoxicity, the decision was made to switch treatment to dapsone.

Due to its inhibition of neutrophil chemotaxis, reduction of reactive oxygen species production, and other pro-inflammatory mediators by neutrophils, dapsone is a useful candidate for diseases characterized by neutrophilic inflammation, such as pyoderma gangrenosum [3,6]. Although the efficacy of dapsone has not been validated in randomized clinical trials, there are several retrospective analyses with favorable outcomes in patients with pyoderma gangrenosum treated with dapsone, showing an improvement in lesions in about 75%-95%, especially with concomitant agents [3,6,7], not only as systemic therapy but also as topic agent showing a favorable response in lesions, with almost 90% of response with a concomitant agent, making this agent a good option for patients who are not candidates for systemic therapy [8,9].

However, similar to azathioprine, myelotoxicity is among the side effects associated with dapsone use, with methemoglobinemia, hemolytic anemia, and agranulocytosis being the most frequently reported effects, with agranulocytosis being a high-risk effect [10]. The coexistence of Felty's syndrome and pyoderma gangrenosum represents a clinical challenge due to the pro-inflammatory state combined with immunosuppression and an increased risk of infections [1,2]. Despite the risk of developing adverse effects, the decision to change the treatment was based on the high risk of associated infections presented by the patient.

Similar to our case, there are several reports of difficult-to-treat PG in which a monoclonal antibody was initiated [11,12]; however, none of them were associated with Felty's syndrome or presented adverse reactions like our case. To our knowledge, there is no study or case report in which dapsone has been used to treat pyoderma gangrenosum in a patient with Felty's syndrome. Dapsone, by increasing neutrophil count and reducing inflammation, offers an integral approach to managing both Felty's syndrome and pyoderma gangrenosum.

## Conclusions

Dapsone represents a viable therapeutic option for the management of pyoderma gangrenosum, particularly in patients who are unresponsive to or intolerant of conventional treatments. Its dual action as an anti-inflammatory and antimicrobial agent, coupled with its relative affordability, reinforces its potential utility in PG treatment regimens. While its use is primarily supported by case studies and series, the existing data suggests significant efficacy in reducing inflammation and promoting ulcer healing. The safety profile of dapsone necessitates careful monitoring, particularly concerning hemolytic anemia and glucose-6-phosphate dehydrogenase (G6PD) deficiency. Further controlled studies are needed to solidify its role in the treatment of PG, but current evidence supports its consideration as a valuable therapeutic option.
